# A convenient tool for gas derivatization using fine-needle capillary mounting for protein crystals

**DOI:** 10.1107/S0909049513021584

**Published:** 2013-10-01

**Authors:** Nobuhiro Mizuno, Masatomo Makino, Takashi Kumasaka

**Affiliations:** a Structural Biology Group, Japan Synchrotron Radiation Research Institute (JASRI/SPring-8), 1-1-1 Kouto, Sayo, Hyogo 6795198, Japan

**Keywords:** crystal mount, xenon, fine-needle capillary, sample changer, cryocrystallography

## Abstract

Gas derivatization of protein crystals is useful not only to analyse gas-binding proteins but also to solve the phase problem of X-ray crystallography by using noble gases. However, the gas pressurization tools for these experiments are often elaborate and need to release the gas before flash-cooling. To simplify this step, a procedure using a fine-needle capillary to mount and flash-cool protein crystals under the pressurization of gases has been developed. After the crystals are picked up with the capillary, the capillary is sealed with an adhesive and then connected directly to a gas regulator. The quality of the diffraction data using this method is comparable with that of data from conventional pressurization procedures. The preparation of xenon-derivatives of hen egg-white lysozyme using this method was a success. In the derivatives, two new xenon binding sites were found and one of their sites vanished by releasing the gas. This observation shows the availability of flash-cooling under gas pressurization. This procedure is simple and useful for preparing gas-derivative crystals.

## Abstract 2

A convenient gas-derivatization tool for protein crystals is presented in combination with a fine-needle capillary and a gas-pressure regulator.

## 1. Introduction

Gas adsorption to proteins is an interesting phenomenon not only as a function of gas-binding proteins but also in the determination of the crystal structures of proteins using noble gases. For many protein crystals, pressurization with the noble gases can be used to introduce a heavy atom into the structure. This method has been shown to be effective for structural determinations (Stowell *et al.*, 1996 ▶; Prangé *et al.*, 1998 ▶). At ambient temperature, diffraction data collections under gas pressurization were initially performed using an original pressure cell (Tilton, 1988 ▶) and by quartz capillary (Schiltz *et al.*, 1994 ▶, 1997 ▶; Stowell *et al.*, 1996 ▶; Quillin & Matthews, 2002 ▶; Colloc’h *et al.*, 2007 ▶). On the other hand, gas-pressurized cryogenic samples have been prepared using a capillary (Schiltz *et al.*, 1997 ▶), an original pressure cell (Soltis *et al.*, 1997 ▶; Hayakawa *et al.*, 2008 ▶) or some commercially available tools: XCell (Oxford Cryosystems, Oxford, UK), Cryo-Xe-Siter (Rigaku, The Woodlands, TX, USA) and Xenon Chamber (Hampton Research, Aliso Viejo, CA, USA), which can be used for the xenon pressurization up to several MPa. However, despite these developments, only about a dozen studies have determined protein structures by noble gases, because the gas pressurization procedure is complicated to handle.

We recently reported a new crystal mounting method that uses a fine-needle capillary (Makino *et al.*, 2012 ▶). With this method, crystals with solvent are pipetted into the capillary, and we flash-cool the capillary and all of its contents. The method is effective for handling and mounting protein microcrystals of less than 10 µm. The scattering of the glass capillary around the mounted crystal is small enough to have no effect on the diffraction data. In addition, the glass capillary is expected to be strong against the gas pressure due to its solid material and tapered structure.

Here we present a new procedure for flash-cooling protein crystals under gas pressurization. This is a simple system to pressurize protein crystals using a fine-needle capillary. The capillary can be easily combined with the automatic sample exchanger SPACE (SPring-8 precise automatic cryo-sample exchanger) (Ueno *et al.*, 2004 ▶). We successfully prepared xenon-binding for hen egg-white lysozyme with this procedure. This procedure may enable the structural determination of proteins with noble gases.

## 2. Materials and methods

### 2.1. Experimental set-up

**Figure 1:**
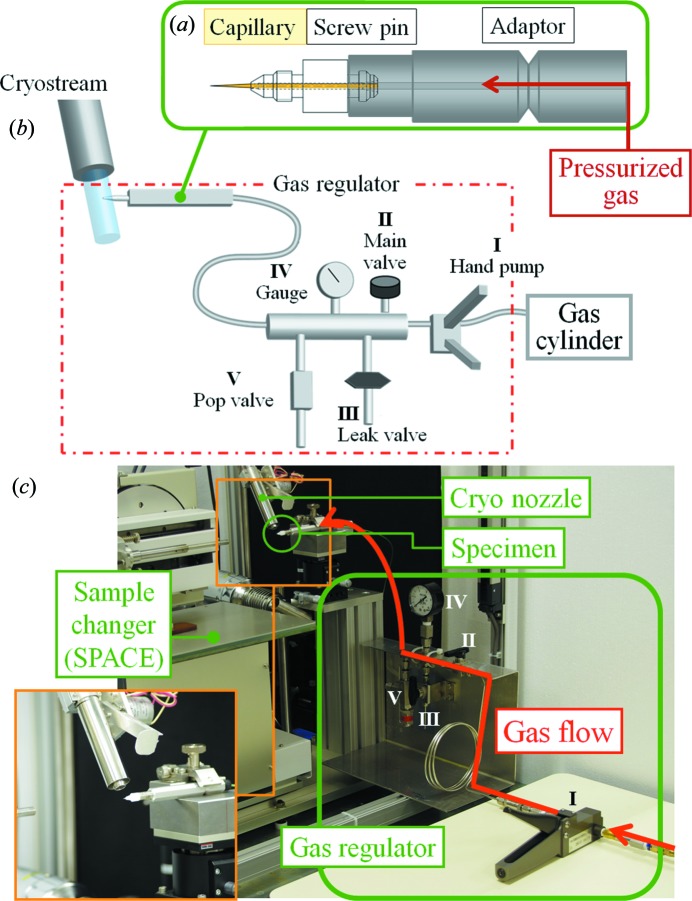
Apparatus for gas derivatization. (*a*) Schematic drawing of the sample pin (white) and adaptor (grey). (*b*) Schematic drawing of the gas regulator attached to the adaptor. (*c*) Photograph of the gas-derivatization system.

The fine-needle capillary to mount crystals was prepared from a glass straight capillary with inner and outer diameters of 0.7 and 1.0 mm, respectively. The fine and tapered tip of the capillary was shaped with a capillary puller (PN-30, Narishige Co., Tokyo) as described previously (Makino *et al.*, 2012 ▶). The diameter of the tip was adjusted to 100 µm using a capillary microforge (MF-900, Narishige Co.). For easier handling and tightening the joints of the gas tubing, the fine-needle capillary was attached to a screw-type sample pin (Rigaku Aihara Seiki Co., Tokyo). This pin is dedicated to SPACE, the SPring-8 automatic sample exchanger (Ueno *et al.*, 2004 ▶), and the capillary was inserted into the inner hole of the pin, which was specially employed for this purpose, as shown in Fig. 1(*a*) ▶.

The sample pin was screwed into the original adaptor for gas injection (Fig. 1*a* ▶) and connected tightly to a gas pressure regulator (Fig. 1*b* ▶). The gas regulator system consists of a main valve for injecting gas into the capillary, and the hand pump of the XCell Pressure Amplifier was used for adjusting the gas pressure (Oxford Cryosystems). To avoid breakage of the capillary, a safety valve was also installed in the system and its maximum pressure was adjusted to 4 MPa, which is the pressure capacity determined from the diameter and the thickness of the capillary using the so-called Barlow’s formula related to the inner pressure of a pipe. The system was integrated with SPACE (Fig. 1*c* ▶) and allowed to flash-cool the capillary in a N_2_ cryostream under pressurization.

SPACE is useful but not essential to this method. After the flash-cooling, a SPACE toolkit including an electric screw driver, adaptors and attachments is useful to transfer the sample to magnetic goniometer head adaptors. The toolkit is also available from Rigaku Aihara Seiki.

### 2.2. Xenon derivatization and data collection of lysozyme crystals

**Figure 2:**
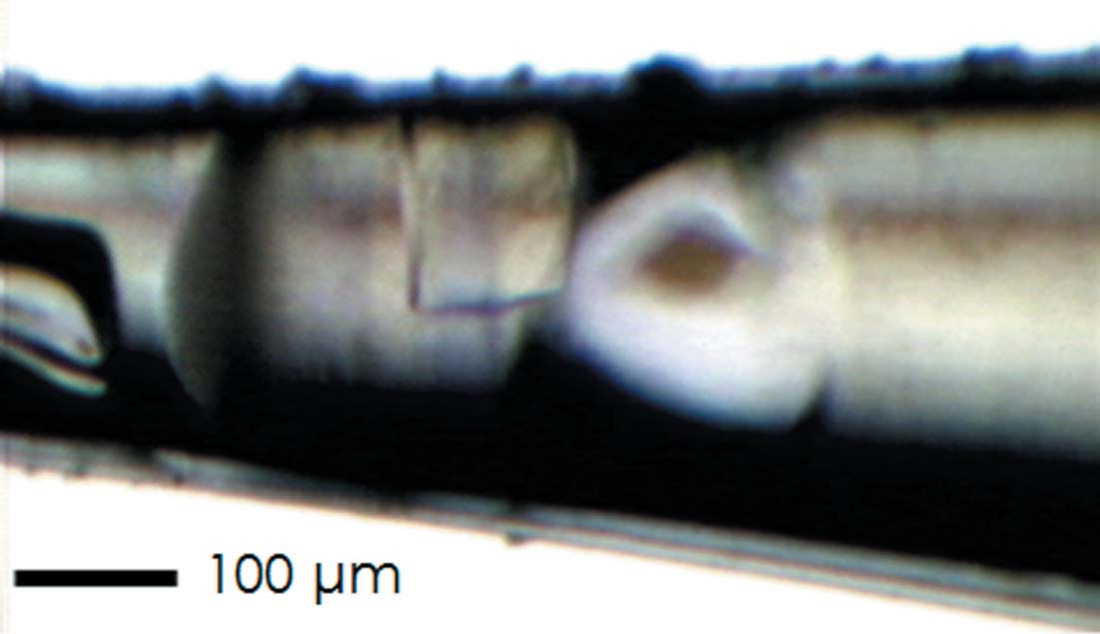
The lysozyme crystal cryocooled under 2 MPa xenon pressurization in a fine-needle capillary.

Hen egg-white lysozyme was crystallized using the hanging-drop vapour-diffusion method as described previously (Steinrauf, 1959 ▶). The tetragonal crystals were soaked for several minutes in cryoprotectant consisting of 50 m*M* acetate buffer (pH 4.5), 1.4 *M* NaCl and 25% (*v*/*v*) glycerol, and then the crystals were pipetted into the fine-needle capillary together with the cryoprotectant solution. As the pipetting method, we examined with a capillary action, a PicoPipet D1 (Altair Co., Tokyo) and a pneumatic microinjector (IM-11-2, Narishige Co.). Although all of the methods attempted were able to introduce the crystals, the current-control type of the PicoPipet D1 was most useful for controlling the suction volume accurately. However, it should be noted that airspace in the fluid channel connected the PicoPipet D1 and the capillary to prevent contamination of the cryoprotectant solution and the liquid media of PicoPipet D1, which is a liquid pump. Then, the tip of the capillary was immediately sealed by cyanoacrylate instant adhesive. The capillary was set to the devices described above and pressurized at 1 or 2 MPa of xenon for 20 min and then flash-cooled at 100 K by a N_2_ cryostream (Fig. 2 ▶). The gas pressure was maintained until SPACE picked up the crystal. To confirm the advantage of flash-cooling under gas pressurization, we also examined flash-cooling after the pressurized gas was released.

Diffraction data of the lysozyme crystals were collected at the SPring-8 beamline BL41XU. All the data collections were conducted at a wavelength of 1 Å using a CCD detector (MX225HE, Rayonix, Evanston, IL, USA). The diffraction images were processed with the *HKL2000* suite (Otwinowski & Minor, 1997 ▶).

We confirmed the presence of anomalous scatterers in all of the crystals by calculating the anomalous difference Fourier map. The phases to calculate the maps were obtained by the atomic model of lysozyme refined with each data set using *REFMAC* (Murshudov *et al.*, 1997 ▶) from the CCP4 suite (Collaborative Computational Project, Number 4, 1994 ▶). The positions of anomalous scatterers were independently solved using *SOLVE* (Terwilliger & Berendzen, 1999 ▶) utilizing the anomalous differences of the data. The occupancies of the xenon sites were refined by *SHELXL* (Sheldrick & Schneider, 1997 ▶). The results are summarized in Table 1 ▶.

## 3. Results and discussion

### 3.1. Gas derivatization using fine-needle capillary

The lysozyme crystals in the fine-needle capillary were successfully flash-cooled by a N_2_ cryostream under the pressurization of xenon gas. The diffraction data of the crystals seem to be comparable with data obtained using conventional cooling methods (Table 1 ▶). The sample pins were compatible with the sample exchanger SPACE, and we can conveniently store large amounts of the crystals and collect diffraction data at SPring-8 macromolecular crystallography beamlines. These techniques (for introducing crystals into the capillary and for flash-cooling) are available based on the premise of using SPACE, and thus the techniques can be used only on SPring-8 sites at this time. However, we are developing an adaptor and connectors of SPACE sample pins so that the techniques can be used on other synchrotrons or in laboratories or with other sample-exchange robots. We also plan to improve the thickness and material of the capillary and adaptor so that high pressure by gases other than xenon can be used.

**Figure 3:**
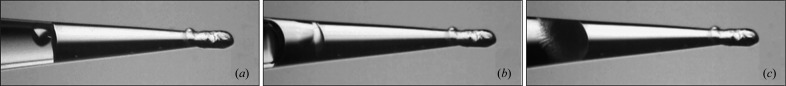
Flash-cooling a crystal under xenon gas pressure. These figures show the crystal in the capillary (*a*) at room temperature, (*b*) just after flash-cooling, and (*c*) a few seconds after flash-cooling.

The diffraction images did not show any ice ring patterns. However, we observed that the filler gas became liquid and then solid during the flash-cooling under pressurization (Fig. 3 ▶). By phase transition of the gas to liquid or solid, the latent heat is released and might cause a thermal change of the materials exposed to the gas. This observation was reported by Schiltz *et al.* (1997 ▶), who suggested that pressurized gas should be released just before flash-cooling to avoid the adverse effect on protein crystals. To avoid this influence under the pressure, we filled the capillary with a large amount of a cryoprotectant solution and kept the crystal off the gas–liquid boundary. As a result, the frozen solution gave ice ring patterns only around the boundary, and the diffraction data of the crystals kept away from the gas by more than 100 µm seemed to be safe (as shown in Table 1 ▶). The solution might work as a thermal barrier absorbing the latent heat, instead of the crystals doing so. However, this possibility needs further investigation.

### 3.2. Xenon binding under pressure

**Figure 4:**
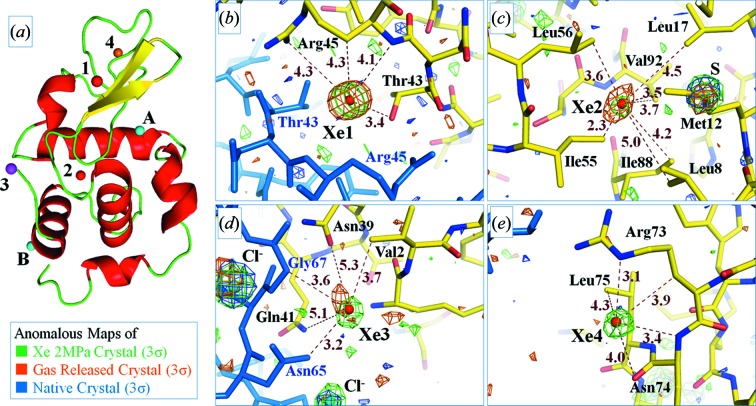
Xenon sites in hen egg-white lysozyme. (*a*) Xe1 and Xe2 are commonly observed sites. Xe3 and Xe4 were newly found in the present study. Xe4 is found only in the crystals cooled under pressurization. The A and B sites were reported previously but not observed in our study. (*b*)–(*e*) Electron density and difference Fourier maps around the xenon sites. Green, orange and blue meshes indicate Bijvoet-difference Fourier using the diffraction data of a crystal under 2 MPa pressurization, a gas released crystal and a native crystal, respectively. Blue sticks indicate symmetry-related molecules. Brown numbers and dots indicate close contacts to the xenon atoms.

In general, xenon is easy to bind to hydrophobic inaccessible cavities of proteins (Prangé *et al.*, 1998 ▶). In two previous studies on the tetragonal lysozyme crystals, four xenon binding sites (Xe1, Xe2, XeA and XeB) were consistently observed to exhibit this property (Prangé *et al.*, 1998 ▶; Takeda *et al.*, 2004 ▶). Among the four sites, Xe1 and Xe2 were common and also found in our study (Fig. 4 ▶). Xe1 is in a molecular surface area surrounded by Thr43 and Arg45, located on a twofold axis in the crystal. Xe2 is located at a hydrophobic core (Leu8, Met12, Leu17, Ile55, Leu56, Ile88 and Val92) in the molecular interior.

The other two sites, XeA and XeB, were not seen in the Prangé *et al.* (1998 ▶) or Takeda *et al.* (2004 ▶) studies and were also not observed in the present study. XeA closely contacts Gln57, Ile58, Ile98, Ala107 and Trp108 (Prangé *et al.*, 1998 ▶), and XeB was located near another twofold axis and surrounded by Ala10, Lys13, Arg14 and Leu129 (Takeda *et al.*, 2004 ▶). The latter sites had lower occupancies or higher thermal factors than those of Xe1 and Xe2; the occupancy and *B*-factor were 0.10 and 36.0 for XeA and 0.32 and 45.3 for XeB, respectively (Table 2 ▶). Thus the latter sites might change the affinity of xenon depending on the experimental conditions such as the composition of the cryoprotectant, the pressure of xenon gas and the temperature employed at data collection.

Two xenon binding sites (Xe3 and Xe4) were newly observed in our study (Fig. 4 ▶). Xe3 is surrounded by the side-chain of Val2, Asn39, Gln41 and the symmetry-related residues of Asn65 and Gly67. Another site Xe4 is located near Arg73, Asn74 and Leu75. Although the new sites are located at solvent-accessible molecular surface areas including polar residues, these occupancies and *B*-factors are comparable with Xe2 and are sufficient to detect the sites in the anomalous difference map (data not shown). In the Xe-2M-R crystal cooled just after gas release, Xe4 could not be found and the *B*-factor of Xe3 increased slightly. The two sites seem to have been found due to flash-cooling under gas pressurization, and thus our method is expected to be more efficient for preparing protein–gas complexes.

## 4. Conclusion

The crystal mounting procedure combined with a fine-needle capillary and a gas pressure system is a convenient way to flash-cool protein crystals under gas pressurization at several MPa. In the xenon-derivatives of tetragonal lysozyme crystals prepared by the method, we found new two xenon binding sites. This simple method efficiently prepares protein–gas complexes, and we are now working to improve the pressure capacity of the system.

## Figures and Tables

**Table 1:** Statistics for data collection and structure determination using xenon-derivatives

	Xe-1M	Xe-2M	Xe-2M-R	Native
Pressure (MPa)	1	2	2 (gas release)	None
Crystal size (µm)	70 × 70 × 30	90 × 90 × 30	80 × 80 × 30	90 × 80 × 30
Cell dimensions (*a*, *c*) (Å)	79.10, 36.98	79.16, 36.99	79.97, 36.96	78.75, 37.31
Resolution (Å)	50.0–1.36 (1.41–1.36)	50.0–1.36 (1.41–1.36)	50.0–1.50 (1.55–1.50)	50.0–1.58 (1.64–1.58)
Completeness	100.0 (99.8)	100.0 (100.0)	100.0 (100.0)	100.0 (100.0)
*I*/σ	57.8 (7.4)	74.4 (12.6)	66.8 (10.2)	63.2 (17.2)
*R*_merge_	0.077 (0.505)	0.074 (0.388)	0.096 (0.516)	0.060 (0.188)
Redundancy	19.2 (16.3)	28.5 (27.9)	28.0 (28.0)	14.1 (14.0)
*R*/*R*_free_ (%)	18.90/20.00	18.53/21.21	19.30/21.31	21.42/23.17
Xe1: occupancy, *B*-factor	0.64, 22.7	0.60, 35.0	0.64, 26.2	–, –
Xe2: occupancy, *B*-factor	0.30, 27.9	0.23, 37.5	0.24, 38.6	–, –
Xe3: occupancy, *B*-factor	0.39, 27.4	0.41, 26.8	0.39, 30.5	–, –
Xe4: occupancy, *B*-factor	0.38, 36.4	0.39, 39.0	–, –	–, –

**Table 2:** Close contacts around the xenon atoms in the binding sites

Atom	Atom (residue)	Distance (Å)
Xe1	Oγ1 (T43)	3.36
	N (R45)	4.05
	Cβ (R45)	4.26
	Nh2 (R45)	4.32
Xe2	Cδ1 (L8)	5.03
	C∊ (M12)	3.48
	Cδ1 (L17)	4.47
	Cδ1 (I55)	2.29
	Cδ1 (L56)	3.57
	Cδ1 (I88)	4.20
	Cγ2 (V92)	3.71
Xe3	Cγ2 (V2)	3.72
	Oδ1 (N39)	5.26
	N∊2 (Q41)	5.12
	Nδ2 (N65)	3.18
	Cα (G67)	3.60
Xe4	Cβ (R73)	3.90
	N∊ (R73)	3.08
	N (N74)	3.37
	O (N74)	3.97
	Cδ2 (L75)	4.28
